# TRPC6 Single Nucleotide Polymorphisms and Progression of Idiopathic Membranous Nephropathy

**DOI:** 10.1371/journal.pone.0102065

**Published:** 2014-07-14

**Authors:** Julia M. Hofstra, Marieke J. H. Coenen, Mascha M. V. A. P. Schijvenaars, Jo H. M. Berden, Johan van der Vlag, Lies H. Hoefsloot, Nine V. A. M. Knoers, Jack F. M. Wetzels, Tom Nijenhuis

**Affiliations:** 1 Department of Nephrology, Radboud university medical center, Nijmegen, the Netherlands; 2 Department of Human Genetics, Radboud university medical center, Nijmegen, the Netherlands; Emory University School Of Medicine, United States of America

## Abstract

**Background:**

Activating mutations in the Transient Receptor Potential channel C6 (TRPC6) cause autosomal dominant focal segmental glomerular sclerosis (FSGS). TRPC6 expression is upregulated in renal biopsies of patients with idiopathic membranous glomerulopathy (iMN) and animal models thereof. In iMN, disease progression is characterized by glomerulosclerosis. In addition, a context-dependent TRPC6 overexpression was recently suggested in complement-mediated podocyte injury in e.g. iMN. Hence, we hypothesized that genetic variants in TRPC6 might affect susceptibility to development or progression of iMN.

**Methods & Results:**

Genomic DNA was isolated from blood samples of 101 iMN patients and 292 controls. By direct sequencing of the entire TRPC6 gene, 13 single nucleotide polymorphisms (SNPs) were identified in the iMN cohort, two of which were causing an amino acid substitution (rs3802829; Pro15Ser and rs36111323, Ala404Val). No statistically significant differences in genotypes or allele frequencies between patients and controls were observed. Clinical outcome in patients was determined (remission n = 26, renal failure n = 46, persistent proteinuria n = 29, follow-up median 80 months {range 51–166}). The 13 identified SNPs showed no association with remission or renal failure. There were no differences in genotypes or allele frequencies between patients in remission and progressors.

**Conclusions:**

Our data suggest that TRPC6 polymorphisms do not affect susceptibility to iMN, or clinical outcome in iMN.

## Introduction

Membranous nephropathy (MN) is the leading cause of nephrotic syndrome in the adult Caucasian population and is characterized by immune complex deposition in the subepithelial layer of the glomerular basement membrane. In one third of patients, MN is caused by an underlying disease such as a malignancy, infection or a systemic disease. Until recently, no underlying cause could be identified in two thirds of patients and, therefore, the disease was considered to be idiopathic (iMN) in these patients. [1] In 2009 Beck *et al*. discovered antibodies against the M-type phospholipase A_2_ receptor (PLA_2_R), a membrane glycoprotein located on the podocyte, in patients with iMN. [2] Further studies showed that antibodies against PLA_2_R are present in approximately 70% of Caucasian patients with active disease. [3], [4] This finding thus provided evidence that iMN is an auto-immune disease.

A recent genome-wide association study showed that genetic variants in *HLA-DQA1* and *phospholipase A2 receptor* (*PLA2R1*) were most significantly associated with biopsy-proven iMN. [5] Association with *HLA* regions has been identified in many autoimmune diseases, the association with the *PLA2R1* gene supports the key role of the anti-PLA2R antibodies in the pathogenesis of the disease. In an additional study we assessed if rare genetic variants within the coding region of the *PLA2R1* gene could explain antibody formation. Although sequencing of the complete coding sequence of *PLA2R1* in 95 patients with iMN confirmed associations of common single nucleotide polymorphisms (SNPs) with iMN, rare variants were present in only a minority of patients (9%). [6] This data questions why iMN is rare, as these SNPs occur frequently in the general population. It could be hypothesized that a rare combination of (relatively) common events (genetic variants and/or environmental factors) might constitute the pathogenetic trigger. In addition to *HLA* and *PLA2R1*, variants in other genes might be involved.

One of the biggest unresolved issues in patients with iMN is the highly variable disease course. Spontaneous remission occurs in about 40–50% of patients, but another 30–50% of patients progresses to end stage renal disease within 10 years. [7] Although several predictive strategies have been proposed, it remains difficult to identify patients with a high risk of disease progression. [8], [9] Progression of disease is accompanied by glomerulsclerosis in renal biopsies. [10]

**Figure 1:**
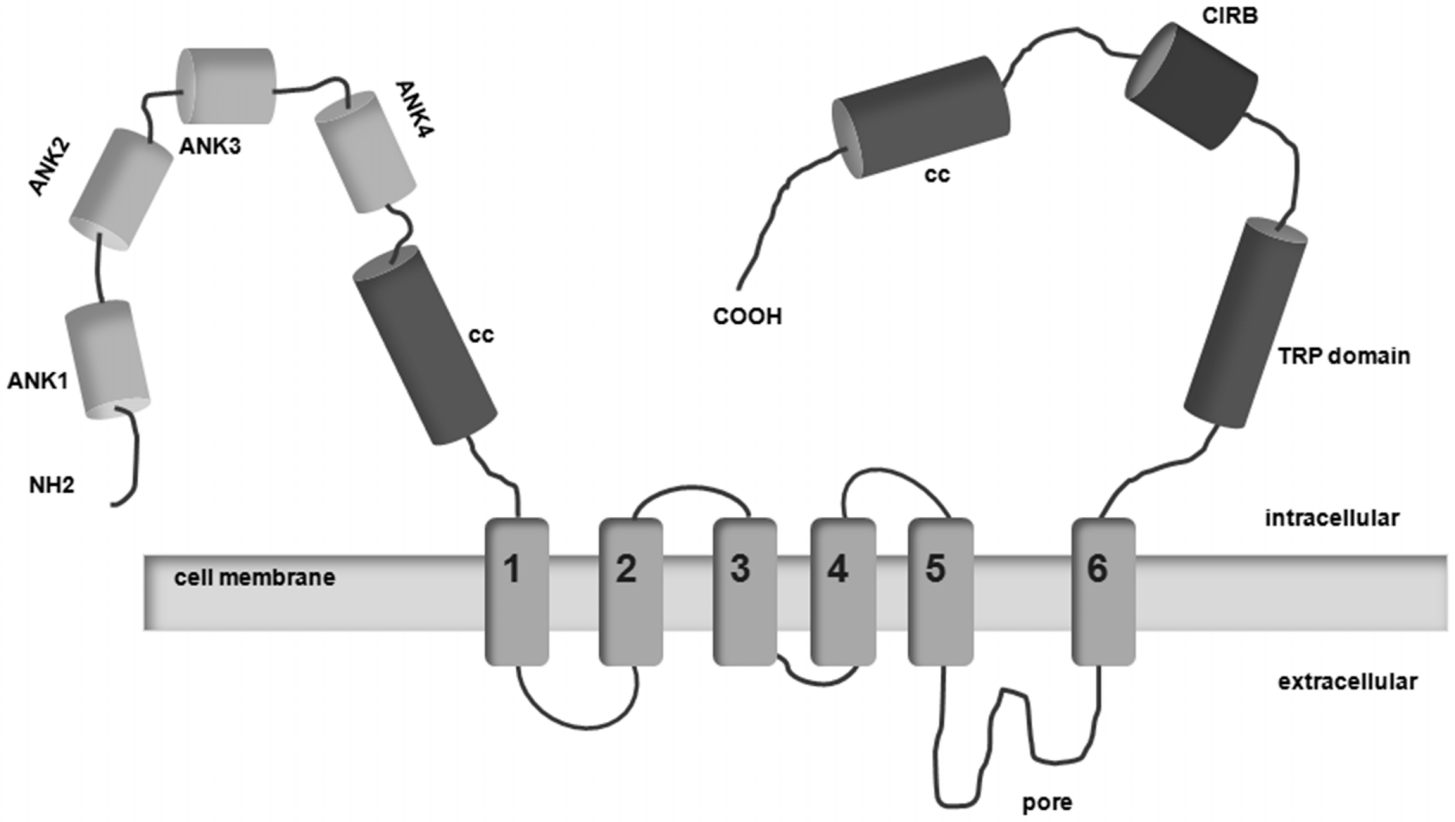
TRPC6 topology. Structure of the TRPC6 monomer. TRPC6 belongs to the large family of TRP channels, which contain six transmembrane domains, one pore-forming region and large intracellular N- and C-tails. Four subunits are required to assemble a functional channel. In the pococyte, TRPC6 is part of the slit diaphragm multiprotein complex. TRP: transient receptor potential, ANK: Ankyrin repeat, cc: coiled–coiled domain, CIRB: CaM/IP3R-binding domain.

The Transient Receptor Potential channel C6 (TRPC6) is an ion channel that is expressed in podocytes at the slit diaphragm {Figure 1}. [11] This complex of interconnected proteins provides both physical linkage as well as a signaling platform that regulates podocyte behavior and architecture, and thereby affects glomerular permeability. TRPC6 was recognized as an important slit diaphragm component after *TRPC6* gain-of-function mutations were shown to be responsible for autosomal dominant focal segmental glomerulosclerosis (FSGS), a leading cause of steroid-resistant nephrotic syndrome that can rapidly progress to end-stage renal disease (ESRD). [11]–[15] Animal studies show that podocyte-specific transgenic TRPC6 overexpression leads to albuminuria and histological findings similar to human FSGS. [14], [16] However, glomerular *TRPC6* expression is also increased in acquired human proteinuric diseases. Importantly, this occurs in human MN, as well as in the passive Heymann nephritis animal model for MN. [13], [17] Furthermore, angiotensin II (AngII) activates specific TRPC6-mediated intracellular pathways in the podocyte. [17]–[20] Angiotensin receptor blockers, angiotensin converting enzyme inhibitors and calcineurin inhibitors, all three utilized to treat proteinuria in MN, were shown to inhibit these TRPC6-mediated cascades. [17], [19] Thus, TRPC6 is not only primarily involved in the pathogenesis of FSGS, but could also mediate podocyte damage and/or progression to ESRD in other proteinuric diseases like MN. Therefore, we hypothesized that sequence variations in the *TRPC6* gene could affect susceptibility to development and/or progression of iMN.

In the present study, we screened a cohort of 101 biopsy-proven iMN patients for *TRPC6* sequence variations by direct sequencing of all 13 exons of the *TRPC6* gene. Furthermore, we assessed whether genetic variants were associated with disease progression and outcome in patients with iMN.

## Subjects and Methods

### Patients

The present study included iMN patients referred to our centre between 1995 and 2007. In all patients, the diagnosis of iMN was established by renal biopsy and secondary causes were excluded according to local routine clinical work up. [21] Baseline data on age, sex and ethnicity were present for all patients. To allow subgroup analysis of anti-PLA_2_R positive patients, the presence of anti-PLA2R antibodies in the serum was evaluated. Serum samples at diagnosis were available for most patients. The assay for measuring anti-PLA_2_R antibodies has been previously described. [22]

iMN patients were evaluated using a standardized protocol as previously described. [23] Patients were prospectively followed, and data on treatment and outcome (e.g. remission and renal survival) are obtained. For the current study we evaluated the occurrence of spontaneous remissions and/or renal failure. Spontaneous remission was defined as proteinuria <2.0 g/day with stable renal function in a patient not treated with immunosuppressive agents. Renal failure/progression was defined as an increase in serum creatinine >25% from baseline with a serum creatinine >135 µmol/l or an increase >50% from baseline.

### Ethics statement

The study was approved by the ethics committee of the Radboud university medical center (Commissie Mensgebonden Onderzoek (CMO) Regio Arnhem Nijmegen, nr.9701-0016). All patients gave written informed consent prior to participation in the study.

### Sequencing

DNA was purified from blood samples using salt extraction according to a previously described protocol. [24] All exons including splice sites in the *TRPC6* gene were sequenced. Sense and antisense primers were designed using Primer3. [25] PCR primers and conditions are given in Table 1. PCR products were purified using Multiscreen filter plates (Millipore Carrigtwohill, Cork, Ireland). Purified products were used for Sanger sequence analysis (bidirectional) with dye-termination chemistry (BigDye Terminator, version 3) on a 3730 DNA analyzer (Applied Biosystems, Inc., Foster City, CA, USA).

**Table 1:** Primers used to sequence the 13 exons of *TRPC6*.

Exon	F/R primer	Primer sequence 5′—3′
1	F primer	GTCTGCCCAGGTCCAGTTC
1	R primer	GTACACACGCGGGTTCAG
2_01	F primer	GATGAATGGCAAGTCATTTGG
2_01	R primer	AACCTCTTGCCTTCAGCAAA
2_02	F primer	GCCAATGAGCATCTGGAAAT
2_02	R primer	AGCCGTCATGACTGGATCTT
2_03	F primer	GATTGAACGGCCTCATGATT
2_03	R primer	GGTAGCGATCACAACTTTTGC
3	F primer	AACGTGGTATTCTCCATTATTGC
3	R primer	AGCACCAACAAGAACCAAAA
4	F primer	GCCATTTGTTTGTTGCCTGT
4	R primer	TGGAGATAAGATTTTTCCCCACT
5	F primer	GGAGATCATTGGAATGTGCAG
5	R primer	AATGAACCCAAGGCAACTGT
6	F primer	TTGGGACCAAATTTTGAAGG
6	R primer	GAGAATTGTGCAGTAACCGAACT
7	F primer	GGAGACTTCCATTCGAAAACC
7	R primer	CCAAAACATTATCCCATGGAC
8	F primer	TCACTAATTTGCAGACACTAAACAA
8	R primer	CGAAGAGCAGTCCATGCTT
9	F primer	CGATCACTGGGGTCTGAGAG
9	R primer	AAAGGGATGTGGCATAGTGG
10	F primer	AGGGAAGAACCCCGTAAGAA
10	R primer	GCTTCTGAACATCTGTCCCTTT
11	F primer	TTGGCAGCCACAAAGTCTAA
11	R primer	AAGAATCACATAGTTCAAGAACCTAAA
12	F primer	GGCTCACTACAGGGAGGAAG
12	R primer	GCTCTCCAGGCACTCTGC
13	F primer	TTTCCTCCTGTCCCACAGTC
13	R primer	GGCTCCAGATGATAGGATGG

F, forward; R, reverse.

### Analysis

*TRPC6* sequences were analyzed for the presence of genetic variants in comparison with the published reference sequence for human *TRPC6* (mRNA NM_004621.5). Ethnically matched population allele frequencies of the identified variants were obtained from the 1000 Genomes project (1000 Genomes release 10 - March 2012). [26] Variants were considered to be common (previously reported with allele frequency >1%), rare (previously reported with allele frequency <1%), or novel (not previously reported in the 1000 Genome project nor in dbSNP 136).

#### SNP genotyping

SNPs that lead to amino acid substitutions, and/or that were rare (previously with allele frequency <1%), completed with SNPs that had a the minor allele frequency in the patient cohort of >10%, were selected for genotyping in 292 healthy individuals from the same geographical region (Table 2). Genotyping was performed using Taqman allelic discrimination assays (Invitrogen, Bleiswijk, The Netherlands) according to the instructions of the manufacturer. The fluorescent signal for allelic discrimination was determined after amplification using an end-point reading on the 7500 Fast Real-time System (Applied Biosystems). Automated allele calling was performed by allelic discrimination plots using SDS 1.4 software (Applied Biosystems). We included 5% duplicate samples in each plate as quality control.

**Table 2:** *TRPC6* variants in patients with iMN (n = 101).

Chr	position	name	ref	All	CEU AF	AF patients	p-value	AF PLA2R	p-value*	ref cDNA	cDNA	prot. level	effect	exon	observations
11	101454244	rs191383391	G	G/T	0.017**	0.025	0.44	0.024	0.56	C	c.-10C>A		5′-ÚTR	1	
11	101454192	rs3802829	G	G/A	0.093**	0.089	0.85	0.113	0.48	C	c.43C>T	p.Pro15Ser	missense	1	NH2-terminal region
11	101453995	rs17096918	C	C/T	0.083	0.069	0.52	0.081	0.93	G	c.170+70G>A		intron		
11	101375549	rs10501986	T	T/C	0.480	0.495	0.71	0.492	0.81	A	c.171-20A>G		intron		
11	101374812	rs144891994	C	C/T	0 ∧	0.005	NA	0.008	NA	G	c.888G>A	p.Thr296Thr	synonymous	2	CCD
11	101374626	rs4542378	G	G/A	0.480	0.490	0.80	0.492	0.81	C	c.945+129C>T		intron		
11	101359750	rs36111323	G	G/A	0.135**	0.165	0.24	0.156	0.52	C	c.1211C>T	p.Ala404Val	missense	4	linker region between CCD & TMD1
11	101347093	rs12366144	A	A/G	0.250**	0.292	0.20	0.347	0.02	T	c.1683T>C	p.Asn561Asn	synonymous	6	linker region between TMD3 & TMD4
11	101342958	rs61743044	G	G/A	0.020	0.020	1.00	0.024	0.75	C	c.2115C>T	p.Tyr705Tyr	synonymous	8	linker region between TMD5 & TMD6
11	101342931	rs144891994	C	C/A	0.008	0.005	0.66	0	0.32	G	c.2142G>T	p.Thr714Thr	synonymous	8	TMD 6
11	101325970	rs7105083	G	G/A	0.263	0.238	0.47	0.266	0.93	C	c.2485-138C>T		intron		
11	101325788	rs72984209	G	G/A	0.077	0.054	0.28	0.081	0.87	C	c.2529C>T	p.Phe843Phe	synonymous	11	
11	101323770	rs12805398	C	C/T	0.140**	0.158	0.49	0.153	0.69	G	c.2712G>A	p.Gln904Gln	synonymous	13	

Chr  =  chromosome; position  =  basepair position based on UCSC genome browser version Human Feb. 2009 (GRCh37/hg19) assembly; name  =  rs identifier; ref  =  genomic reference allele; all  =  alleles; CEU AF  =  allele frequencies variant allele in control population. Control population consists of Caucasian population from 1000 Genomes project (release 10 - March 2012), complemented with data form 292 geographically matched controls for variants marked with **; ∧ =  not reported in 1000 Genomes project; AF patient  =  patient minor allele frequencies; p-value  =  comparison allele frequencies controls and patients; AF PLA2R  =  minor allele frequencies in PLA2R positive patients (n = 62); p-value*  =  comparison allele frequencies controls and PLA2R positive patients; genomic level  =  NC_000002.11; ref cDNA  =  reference allele cDNA (given that TRPC6 is on the negative strand); cDNA  =  NM_004621.5; prot. level  =  NP_004612.2; CCD  =  coiled-coiled domain; TMD  =  transmembrane domain.

#### Association analysis

Frequencies of SNPs identified in iMN patients were compared with genotype data of 379 Caucasian controls that were downloaded from the 1000 Genomes project (release 10). In an attempt to increase power, data of 292 healthy controls were added for variants in 5 SNPs, as detected with Taqman assay. Association analyses were performed using the whole genome analysis toolset PLINK (version 1.07). [27] Significance was set a p<0.05. SNPs with an allele frequency of 0.1 and higher were used to construct haplotypes. Haplotype analysis was also performed using PLINK. Next, frequencies of SNPs identified in patients with progressive disease (renal failure) were compared with frequencies observed in patients with a spontaneous remission of proteinuria as described above.

#### Power calculations

In order to be able to estimate the effect size of the genetic variants, we performed a genetic power calculation using the online Harvard Genetic Power Calculator (http://pngu.mgh.harvard.edu/~purcell/gpc/). We calculated the effect size that could be excluded with 80% power and a two-sided p-value of 0.05, for various frequencies of the high-risk allele (ranging from 0.200 to 0.008).

## Results

### Patient characteristics

A total of 101 patients with biopsy proven iMN were included in the present study. Patients were mostly male (77%) and mean age at diagnosis was 51±15 years. All patients were of self-reported Caucasian ancestry. Serum samples at onset of disease were available for 92 patients, anti-PLA_2_R antibodies were present in 62 samples (67%).

### Sequencing

In 101 patients with iMN we identified eight sequence variants in the coding region of the *TRPC6* gene (Table 2). Of the eight coding variants observed, six were common SNPs and two were rare (See Methods for definitions). These rare variants (c.888G>A and c.2142G>T) were both encountered once in two different patients in a heterozygous state, but did not result in amino acid changes (synonymous mutations). Only two of the common SNPs resulted in an amino acid substitution (rs3802829; Pro15Ser and rs36111323, Ala404Val). Four additional common SNPs were observed in the intron regions, as well as one rare variant in the 5′-UTR (c1-10 C>A). The latter was present in 5 patients, all in a heterozygous state.

### Association between *TRPC6* variants and occurrence of iMN

Control data were obtained from the 1000 Genomes project as described. To increase power to detect possible genotype frequency differences between our iMN cohort and the general population, 292 healthy Caucasian controls from the same geographical area were included as an additional control group for the frequency of five SNPs. These five SNPs were selected by previously specified criteria. Association analysis showed that there were no significant differences in the allele frequencies of both the coding and non-coding SNPs between the 101 iMN patients and ethnically matched controls (Table 2). Based on our power calculations, the effect sizes that could be excluded for the various frequencies of the high-risk allele were above 1.6 to 4. For the two SNPs leading to amino acid substitutions in exons, these were 1.7 and 1.8, respectively.

When analyzing associations for the subgroup of PLA_2_R positive patients (n = 62), the synonymous SNP rs12366144 was more frequently present in patients than in controls (p = 0.02) (Table 2). However, when correcting for the multiple testing of 12 SNPs, (p-value should be less than 0.05/12 = 0.00417), this significant association disappeared. Haplotype analysis was performed including all available SNPs, but no significant associations were determined.

### Association between *TRPC6* variants and clinical outcome

After a median follow-up of 80 months (range 15–166) spontaneous remission had occurred in 26 patients, and 46 patients had progressed to renal failure. Of the remaining patients, six had persistent proteinuria >2.0 gram/day with stable renal function, 19 patients were censored for outcome analysis because of treatment with immunosuppressive agents, and four patients were lost to follow-up. None of the 13 identified *TRPC6* sequence variations correlated with remission or renal failure (Table 3). An additional analysis comparing those patients in whom spontaneous remission occurred (n = 26) to all others (n = 75) also did not show differences between groups (data not shown).

**Table 3:** Genotypes in iMN patients with clinical remission versus patients with renal failure.

	Genotype	Remission n = 26 (%)		Renal Failure n = 46 (%)		p-value*
rs191383391	CC	25	(96.2)	43	(93.5)	0.64
	CA	1	(3.8)	3	(6.5)	
rs3802829	CC	22	(84.6)	37	(80.4)	0.68
	CT	4	(15.4)	9	(19.6)	
rs17096918	GG	23	(88.5)	37	(80.4)	0.40
	GA	3	(11.5)	9	(19.6)	
rs10501986	AA	9	(34.6)	10	(21.7)	0.09
	AG	15	(57.7)	25	(54.3)	
	GG	2	(7.7)	11	(23.9)	
rs144891994	GG	26	(100)	46	(100)	1.00
rs4542378	CC	9	(34.6)	9	(19.6)	0.09
	CT	15	(57.7)	27	(58.7)	
	TT	2	(7.7)	10	(21.7)	
rs36111323*	GG	19	(73.1)	31	(67.4)	0.74
	GA	7	(26.9)	14	(30.4)	
rs61743044	CC	26	(100)	43	(93.5)	1.00
	CT	0	(0)	3	(6.5)	
rs12366144	TT	16	(61.5)	20	(43.5)	0.18
	TC	9	(34.6)	23	(50.0)	
	CC	1	(3.8)	3	(6.5)	
rs145077205	GG	25	(96.2)	46	(100)	0.18
	GT	1	(3.8)	0	(0)	
rs7105083	CC	15	(57.7)	26	(56.5)	0.91
	CT	10	(38.5)	18	(39.1)	
	TT	1	(3.8)	2	(4.3)	
rs72984209	CC	23	(88.5)	41	(89.1)	0.93
	CT	3	(11.5)	5	(10.9)	
rs12805398	GG	18	(69.2)	32	(69.6)	0.88
	GA	8	(30.8)	14	(30.4)	

*N = 45 for renal failure.

*P-value for minor allele frequencies-comparison (Mann-Whitney U).

## Discussion

In this study, 13 SNPs in the gene encoding *TRPC6* were identified in our cohort of 101 biopsy-proven iMN patients. None of the identified SNPs was associated with the occurrence of iMN *per se*. In addition, the presence of *TRPC6* SNPs was not associated with clinical outcome in terms of remission rate, persistence of proteinuria or progression to renal failure. Thus, our data do not confirm the hypothesis that *TRPC6* SNPs could confer susceptibility to, or alter the clinical course in iMN.

Pathogenesis of iMN has been an unsolved mystery for decades. The histological features of MN were first described in 1957. [28] The characteristic finding in kidney biopsies of subepithelial deposits that consisted of IgG and complement pointed to an immunological disease ontology. In a rat model of MN, Heymann's nephritis, the disease is caused by IgG antibodies targeting megalin, a protein expressed by both rat tubular cells and podocytes. [29] Since megalin is not present on human podocytes, other pathophysiological mechanisms and other antigenic targets have been considered. A major breakthrough came with the discovery by Beck *et al*. of circulating auto-antibodies against the M-type Phospholipase A_2_ receptor (PLA2R), a protein expressed by human podocytes, in the majority of patients with iMN. [2] Anti-PLA2R antibodies in iMN are mainly of the IgG4 subclass, both in serum and in biopsies. IgG4 is considered not to activate complement, but immune deposits in iMN contain large amount of complement C3. It has been suggested that anti-PLA2R antibodies might activate the lectin pathway of the complement cascade by binding to Mannose Binding Lectin. [30] However, the mechanism by which the antibodies finally lead to the membranous phenotype with severe podocyte damage is still unknown. Furthermore, the trigger for autoantibody formation remains unclear. A rare combination of (relatively) common events (genetic variants and/or environmental factors) might serve as a pathogenetic trigger in a multi-hit setting.

Next to these remaining questions on pathogenetic issues, the substantial interindividual variability in the course and prognosis of the iMN is also unexplained as yet. Why is the disease benign with a single episode and spontaneous remission in some patients, and do other patients progress to ESRD after several treatment resistant episodes? The genetic profile of the patient may be one of the aspects contributing to this interindividual variability. In another glomerulopathy, IgA nephropathy, common polymorphisms in several genes (*MYH*, *TLR9*) were associated with disease progression and development of ESRD. [31], [32] Since *TRPC6* gain-of-function mutations cause podocyte injury leading to hereditary FSGS, glomerular TRPC6 expression is increased in MN, and glomerulosclerosis occurs in progressive MN, we hypothesized that polymorphisms in *TRPC6*, contribute to the susceptibility to develop iMN and/or progress to end-stage renal disease. Of note, Chen *et al*. previously showed that three *TRPC6* SNPs, which they selected on reasons that were not clearly defined, did not correlate with the occurrence of MN. [33] In our study, all exons were sequenced including splice sites in the *TRPC6* gene to identify polymorphisms in the coding regions of *TRPC6* in an unbiased manner. While we identified *TRPC6* SNPs in iMN patients, we found no association of these SNPs with disease occurrence or progression, although based on the number of patients in our cohort a limited effect size could have been missed. This means that the alleles we identified could still have functional effects on TRPC6 channel activity, of which the resulting clinical effect would fall under the effect size we can rule out. However, the majority of the identified SNPs [11] were either intronic or synonomous, minimizing the chance of any functional relevance. The two SNPs causing predicted amino acid substitutions, although of interest, clearly did not contribute to the development of MN in all patients.

In the PLA2R positive patients, one SNP showed an association, but the statistical significance of this association was lost after correction for multiple testing. In terms of genetic association studies, our cohort is quite small and we, therefore, cannot exclude that our study is underpowered to find existing associations, especially with regard to haplotypes. As only the coding regions of the *TRPC6* gene were sequenced, information on intronic polymorphisms and/or SNPs in the promoter region of the gene that might influence expression of the TRPC6 protein, is lacking. Since it is *TRPC6* gain-of-function that is associated with podocyte injury, transcriptionally-mediated increased or diminished *TRPC6* expression as a result of genetic variants in *TRPC6* regulatory regions, might influence susceptibility and prognosis.

However, assuming that *TRPC6* SNPs do not affect occurrence and progression in iMN, the question still remains why TRPC6 is overexpressed in iMN. Since TRPC6 is overexpressed in several human glomerular diseases and animal models of podocyte injury and glomerular disease, it could be a common effector in the resulting podocyte injury.[13], [17] We have previously shown that AngII-mediated activation of the deleterious calcineurin/NFAT pathway, which includes Ca^2+^ influx through TRPC6 itself, leads to enhanced transcriptional expression of TRPC6 in the podocyte. [17] In addition, antiproteinuric agents like angiotensin receptor blockers, angiotensin converting enzyme inhibitors, calcineurin inhibitors and vitamin D normalize TRPC6 expression in podocyte injury and experimental glomerular disease. [17], [34] Alternatively, an interesting hypothesis could be that e.g. anti-PLA2R antibodies actually activate PLA2R on the surface of the podocyte. In cell types other than podocytes, Ca^2+^ influx was demonstrated to be coupled to PLA2R activation. [35] Hypothetically, binding of anti-PLA2R antibodies to PLA2R on the podocyte could activate Ca^2+^ influx through TRPC6 and/or increase TRPC6 expression. A recent study from Kistler et al.,suggests that increasing TRPC6 expression might at first actually serve to protect the podocyte from complement-mediated injury, which seems crucial in MN. [36] Eventually, prolonged TRPC6 overexpression might then become deleterious. While *TRPC6* SNPs do not appear to alter susceptibility to iMN occurrence and progression, this new hypothesis suggests that TRPC6 indeed plays a pathophysiological role in iMN.
